# Use of the multiple-locus variable-number tandem repeat fingerprinting method and microfluidic chips for rapid genotyping of *Staphylococcus aureus* isolates

**DOI:** 10.1186/1753-6561-5-S6-P177

**Published:** 2011-06-29

**Authors:** AJ Sabat, M Chlebowicz, H Grundmann, W Postma, W Baas, J Arends, G Kampinga, N Meessen, AW Friedrich, JM van Dijl

**Affiliations:** 1 Medical Microbiology, University Medical Center Groningen, Groningen, Netherlands

## Introduction / objectives

The early detection of outbreaks of methicillin-resistant *Staphylococcus aureus* (MRSA) and a rapid and accurate identification of sources and routes of transmission are important objectives in hospital settings. In this study we investigated the application potential of Multiple-Locus Variable number tandem repeat Fingerprinting (MLVF) combined with microfluidic technology for a rapid discrimination of MRSA lineages in outbreak settings.

## Methods

A total of 206 non-repetitive MRSA isolates recovered from infected patients at the University Medical Center Groningen between 2000 and 2010 were tested. The results obtained by MLVF using microcapillary electrophoresis were compared to those obtained by *spa* typing and Multiple-Locus Variable number tandem repeat Analysis (MLVA).

## Results

The discriminatory power was 0.98 (107 patterns), 0.969 (85 allelic profiles) and 0.959 (66 types) for MLVF, MLVA and *spa* typing, respectively. Isolates defined as identical by MLVF were almost always (99%) indistinguishable by *spa* typing. All methods tested showed a high concordance of results calculated by adjusted Rand's coefficients. Of the three tested methods, MLVF is the cheapest, fastest and easiest to perform.

## Conclusion

MLVF applied to microfluidic polymer chips is a rapid, cheap, reproducible and highly discriminating tool to determine the clonality of MRSA isolates, and to trace the spread of MRSA strains over periods of many years. Although *spa* typing should be used due to its portability of data, MLVF has a high added-value because it is more discriminatory.

## Disclosure of interest

None declared.

